# Calcium Channel Blocker Overdose

**DOI:** 10.21980/J8CQ07

**Published:** 2024-01-31

**Authors:** Jessica G Andrusaitis, Alan Givertz

**Affiliations:** * Sutter Roseville Medical Center, Department of Emergency Medicine, Roseville, CA; ^ St Agnes Medical Center, Department of Emergency Medicine, Fresno, CA

## Abstract

**Audience:**

Emergency medicine residents and medical students on emergency medicine rotation.

**Background:**

Calcium channel blocker (CCB) overdoses can be severe with potentially serious adverse outcomes. CCBs work by blocking the calcium channels on smooth and cardiac muscle tissue. At low dose ranges, dihydropyridine CCBs (such as nifedipine, amlodipine, and nicardipine) block the L-type calcium receptors in the peripheral vasculature, whereas non-dihydropyridine CCBs (such as: verapamil and diltiazem) affect the L-type calcium receptors in the myocardium.^1^ Because of this distinction, dihydropyridine CCB toxicity manifests as arterial vasodilation and non-dihydropyridine CCB toxicity is associated with cardiac manifestations such as bradycardia and negative inotropy.^2^ It is important to note that in high concentrations (such as in overdoses), CCBs lose specificity for their specific receptors and can show all the manifestations of toxicity such as bradycardia, peripheral vasodilation, and hypotension. Patients can develop both vasoplegic shock from peripheral vasodilation and cardiogenic shock. This is a high acuity low occurrence case with infrequently used but specific treatments, and thus this case provides educational value.

**Educational Objectives:**

At the end of this oral board session, examinees will: (1) demonstrate ability to evaluate a patient with undifferentiated shock with bradycardia and discuss the differential diagnosis, (2) recognize the signs and symptoms of calcium channel blocker overdose, (3) demonstrate ability to manage treatment of a patient with calcium channel overdose.

**Educational Methods:**

This oral board case followed the standard American Board of Emergency Medicine-style case in a tertiary care hospital with access to all specialists and resources needed. This case was tested using 12 resident volunteers ranging from PGY 1–2 in an ACGME (Accreditation Council for Graduate Medical Education) accredited emergency medicine residency program.

**Research Methods:**

Immediate feedback was solicited both from the learners and from the evaluators following the debriefing session. Residents were asked to evaluate the educational value of the case using a 1–5 Likert scale (5 being excellent). Evaluators were asked to score the residents using the ACGME core competencies with a scale of 1–8, 1–4 being unacceptable and 5–8 being acceptable.

**Results:**

Seven PGY1 residents and five PGY2 residents, thus twelve residents in total, completed the case. The average score was 5.10/8. Three residents missed zero critical actions. The most common critical action missed was consulting cardiology or cardiothoracic surgery for circulatory support options. Many residents failed to recognize that the patient did not have a perfusing blood pressure at the beginning of the case and did not start CPR. Although most residents recognized the patient’s hemodynamic collapse was from a calcium channel blocker overdose, most did not know the treatment for this beyond atropine and intravenous fluids.

The learners rated the educational value of the case as 4.9/5. Seven residents reported that the case definitely increased their medical knowledge; five residents reported that it somewhat increased their medical knowledge. All residents rated the case as helpful in preparing to manage this medical condition.

**Discussion:**

The educational content from this case was effective. This is a high acuity low occurrence case that has unique treatments that are not commonly used. This makes this case excellent for practice and discussion. We learned during implementation that this case has a high degree of difficulty compared to other cases, and junior learners will need more prompting. It is also important for the proctor to keep the case moving because there is a lot to cover in the allotted amount of time.

**Topics:**

Calcium channel blocker overdose, toxicology.

## USER GUIDE

[Table t1-jetem-9-1-o1]

**Table t1-jetem-9-1-o1:** 

List of Resources:	
Abstract	1
User Guide	3
For Examiner Only	4
Oral Boards Assessment	10
Stimulus	13
Debriefing and Evaluation Pearls	25

**Learner Audience:**

Interns, junior residents, senior residents

**Time Required for Implementation:**

Case: 15 minutes

Debriefing: 10 minutes

**Learners per instructor:**

1 Learner

**Topics:**

Calcium channel blocker overdose, toxicology.

**Objectives:**

By the end of this practice oral boards case, the learner will:

1. Demonstrate ability to evaluate a patient with undifferentiated shock with bradycardia and discuss the differential diagnosis.
2. Recognize the signs and symptoms of calcium channel blocker overdose.
3. Demonstrate ability to manage treatment of a patient with calcium channel overdose. 4.

### Linked objectives and methods

This oral boards case allows learners to obtain first-hand simulated experience diagnosing and managing a patient with a calcium channel blocker overdose. This oral boards case allows learners to evaluate a patient with undifferentiated shock and bradycardia, review the differential diagnoses, and appropriately diagnose and manage a calcium channel blocker overdose patient (objectives 1–3).

### Recommended pre-reading for instructor

- None, review references as needed

### Results and tips for successful implementation

The case was initially presented as oral board case in a small group setting with approximately 20 emergency medicine residents. We devised a schedule that allowed for each resident to have 30 minutes with each proctor that included 15 minutes for doing the case, 10 minutes for debrief, and a final 5 minutes for scoring the resident. We received positive feedback from residents who found the case useful and interesting. The case was difficult for novice learners, but more straightforward for advanced learners. Novice learners will require more prompting to manage the patient appropriately. We suggest this case should not be tested on interns who do not have experience with oral boards cases. This case initially was presented as a CCB overdose with concomitant ACE inhibitor overdose, but after implementation, we realized the case was difficult enough with just the CCB and thus decided to eliminate the ACE inhibitor component of the case. While this case is best done as an oral boards case, it can also be done as a simulation case or small group session. It is important to debrief the case with the learner after completion of the case or provide post-case reading material.

## Figures and Tables

**Table t2-jetem-9-1-o1:** 

**Vitals:** HR 26	BP not able to obtain	RR 8	O_2_Sat 88% room air
Temp 37°C (98.9°F) (Rectal temperature: 37.5°C, 99.6°F)			

**Table t3-jetem-9-1-o1:** 

	Milestone	Did not achieve level 1	Level 1	Level 2	Level 3
1	**Emergency Stabilization (PC1)**	□ **Did not achieve Level 1**	□ **Recognizes abnormal vital signs**	□ **Recognizes an unstable patient, requiring intervention** **Performs primary assessment** **Discerns data to formulate a diagnostic impression/plan**	□ **Manages and prioritizes critical actions in a critically ill patient** **Reassesses after implementing a stabilizing intervention**
2	**Performance of focused history and physical (PC2)**	□ **Did not achieve Level 1**	□ **Performs a reliable, comprehensive history and physical exam**	□ **Performs and communicates a focused history and physical exam based on chief complaint and urgent issues**	□ **Prioritizes essential components of history and physical exam given dynamic circumstances**
3	**Diagnostic studies (PC3)**	□ **Did not achieve Level 1**	□ **Determines the necessity of diagnostic studies**	□ **Orders appropriate diagnostic studies** **Performs appropriate bedside diagnostic studies/procedures**	□ **Prioritizes essential testing** **Interprets results of diagnostic studies** **Considers risks, benefits, contraindications, and alternatives to a diagnostic study or procedure**
4	**Diagnosis (PC4)**	□ **Did not achieve Level 1**	□ **Considers a list of potential diagnoses**	□ **Considers an appropriate list of potential diagnosis** **May or may not make correct diagnosis**	□ **Makes the appropriate diagnosis** **Considers other potential diagnoses, avoiding premature closure**
5	**Pharmacotherapy (PC5)**	□ **Did not achieve Level 1**	□ **Asks patient for drug allergies**	□ **Selects an appropriate medication for therapeutic intervention, considering potential adverse effects**	□ **Selects the most appropriate medication(s) and understands mechanism of action, effect, and potential side effects** **Considers and recognizes drug-drug interactions**
6	**Observation and reassessment (PC6)**	□ **Did not achieve Level 1**	□ **Reevaluates patient at least one time during the case**	□ **Reevaluates patient after most therapeutic interventions**	□ **Consistently evaluates the effectiveness of therapies at appropriate intervals**
7	**Disposition (PC7)**	□ **Did not achieve Level 1**	□ **Appropriately selects whether to admit or discharge the patient**	□ **Appropriately selects whether to admit or discharge** **Involves the expertise of some of the appropriate specialists**	□ **Educates the patient appropriately about their disposition** **Assigns patient to an appropriate level of care (ICU/Tele/Floor)** **Involves expertise of all appropriate specialists**
22	□ **Patient centered communication (ICS1)**	□ **Did not achieve level 1**	□ **Establishes rapport and demonstrates empathy to patient (and family)** **Listens effectively**	□ **Elicits patient’s reason for seeking health care**	□ **Manages patient expectations in a manner that minimizes potential for stress, conflict, and misunderstanding.**
23	□ **Team management (ICS2)**	□ **Did not achieve level 1**	□ **Recognizes other members of the patient care team during case (nurse, techs)**	□ **Communicates pertinent information to other healthcare colleagues**	□ **Communicates a clear, succinct, and appropriate handoff with specialists and other colleagues** **Communicates effectively with ancillary staff**

**Table t4-jetem-9-1-o1:** 

**#1**	**Patient Information Form**
**#2**	**Rhythm strip**
**#3**	**12 Lead EKG**
**#4**	**Portable chest xray**
**#5**	**Portable chest xray after intubation**
**#6**	**Complete blood count**
**#7**	**Chemistry panel**
**#8**	**Troponin and BNP**
**#9**	**Venous blood gas and lactate**
**#10**	**Alcohol, salicylate, and acetaminophen levels**
**#11**	**Urine drug screen**

**Table t5-jetem-9-1-o1:** 

**Patient’s Name:**	**Jay Cloverfield**	
**Age:**	**29 years**	
**Gender:**	**Male**	
**Chief Complaint:**	**Altered mental status**	
**Person Providing History:**	**EMS**	
**Vital Signs:**		
**Temp:**	**37°C (98.9 ºF) (Rectal temperature)**	
**BP:**	**not obtainable**	
**P:**	**26**	
**RR:**	**8**	
**Pulse Ox:**	**88% on 15 liters nonrebreather**	
**Weight:**	**80.2 kg ( lbs)**	**Height: 70 inches**

**Figure f1-jetem-9-1-o1:**
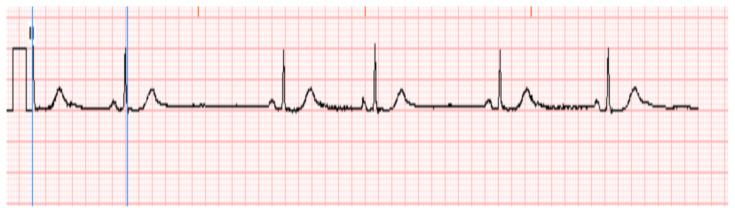


**Figure f2-jetem-9-1-o1:**
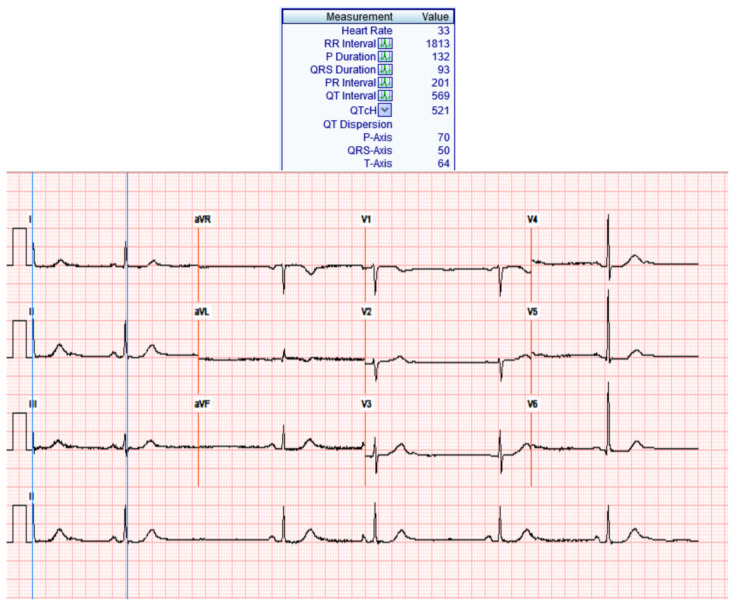


**Figure f3-jetem-9-1-o1:**
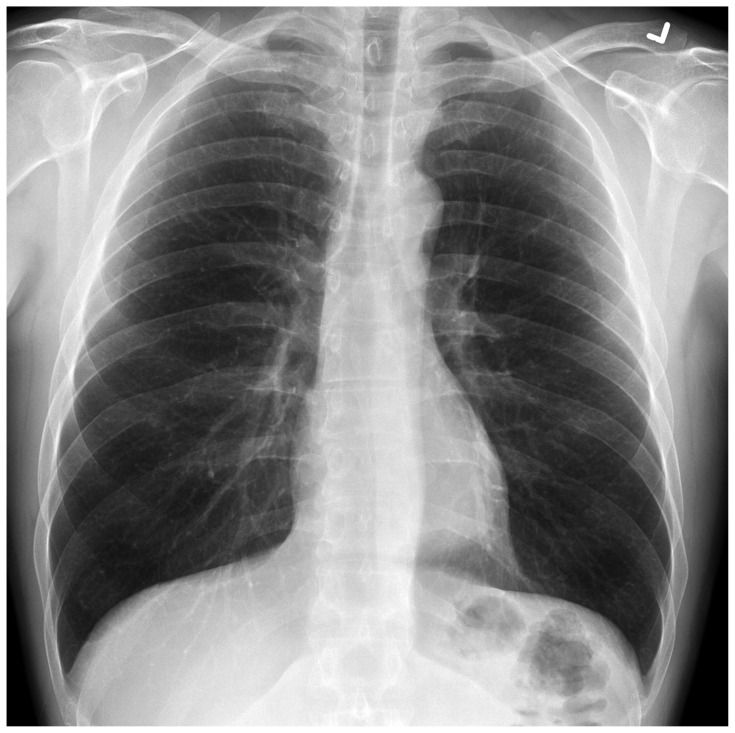


**Figure f4-jetem-9-1-o1:**
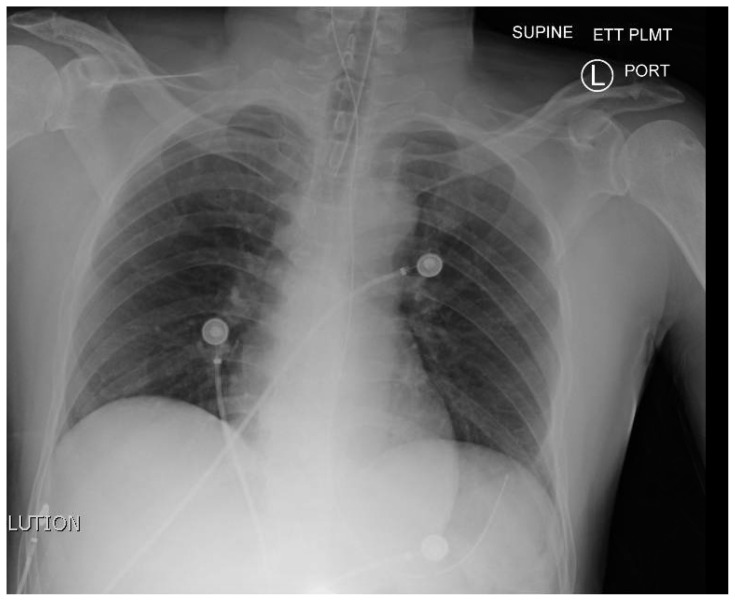


**Table t6-jetem-9-1-o1:** 

**White blood cell count (WBC)**	**9.2 ×1000/mm3**
**Hemoglobin (Hgb)**	**13.1 g/dL**
**Hematocrit (Hct)**	**40.5%**
**Platelets**	**220 ×1000/mm3**
**Differential Neutrophils**	**68%**
**Bands**	**0%**
**Lymphocytes**	**25%**
**Monocytes**	**5%**
**Eosinophils**	**1%**
**Basophils**	**1%**

**Table t7-jetem-9-1-o1:** 

**Sodium**	**135 mEq/L (L)**
**Potassium**	**5.0 mEq/L**
**Chloride**	**95 mEq/L**
**Bicarbonate**	**8 mEq/L**
**Blood Urea Nitrogen (BUN)**	**20 mg/dL**
**Creatinine (Cr)**	**0.7 mg/dL**
**Glucose**	**110 mg/dL**

**Table t8-jetem-9-1-o1:** 

**Troponin - I (NG/ML)**	**110 ng/L**	**(Reference: < 12 ng/L – normal 12 ng/L – 81 ng/L – indeterminant > 81 ng/L – elevated)**
**BNP (pg/ml)**	**200 pg/ml**	**(Reference: < 100 pg/ml – normal)**

**Table t9-jetem-9-1-o1:** 

**pH**	**7.15**
**pCO2**	**63 mmHg**
**pO2**	**30 mmHg**
**Bicarb**	**8 mmol/L**
**Lactate**	**7.2 mmol/L**

**Table t10-jetem-9-1-o1:** 

**ETOH**	**0.8 (80 mg/100 ml)**
**Salicylate**	**Negative**
**Acetaminophen**	**Negative**

**Table t11-jetem-9-1-o1:** 

**Amphetamine**	**Negative**
**Barbiturates**	**Negative**
**Benzodiazepines**	**Negative**
**Cannabinoid**	**Negative**
**Opioid**	**Negative**
**Cocaine**	**Negative**
**Phencyclidine**	**Negative**

## FOR EXAMINER ONLY

### Oral Case Summary

**Diagnosis: Calcium Channel Blocker Overdose**

**Case Summary:** The patient is a 29-year-old male, Mr. C, who is brought from home by the paramedics after his mother found him confused with some empty pill bottles nearby. The patient was found in bed, and there does not appear to be any trauma. He was found at around 7 AM; his mother had last seen him well last night at 9 PM. Paramedics bring empty bottles of amlodipine. The bottles were just filled three days ago and the prescription was for 90 days. The bottles are empty. Mother denies that the patient had had any recent illnesses. No further history is obtainable from the patient.

**Order of Case:** The patient arrives on a gurney to the ED, is receiving oxygen through a 15 L nonrebreather and has one IV in place. The learner performs the ABC’s. The learner recognizes that the patient is completely unresponsive, has heart rate of 26 on the monitor but no palpable pulses, and the nurses cannot get a blood pressure. The learner immediately requests that compressions be started and intubates the patient. ACLS begins. The candidate requests cardiac monitor, pulse oximeter, second large bore IV, and asks that blood be drawn to hold for laboratory evaluation. The candidate should give an IVF bolus. Atropine is given with no response. Epinephrine and calcium are given IV with a return of blood pressure and palpable peripheral pulses, but this only lasts for a few minutes after administration. The epinephrine and calcium can be repeated but the response is still the same: there is a short-term improvement but the patient becomes bradycardic and hypotensive again. The patient continues to have electrical activity but cannot maintain perfusion without starting a vasopressor drip. The vasopressor drip is hung and the patient does not require compressions but is still bradycardic and has a blood pressure of about 80 systolic.

The candidate should order a laboratory evaluation to include CBC, CMP, troponin, BNP, VBG, urine toxicology screen, alcohol level, salicylate level, acetaminophen level, and a serum osmolarity. ECG and CXR should also be ordered. A head CT should also be considered given the altered mental status; however, patient is currently too unstable to leave the department for that.

The learner should contact Poison Control, start high-dose insulin and lipid emulsion. The blood pressure remains poor; the learner should add a second vasopressor and can consider inserting a pacemaker to obtain a pulse rate over 60. In spite of all the medications and maximum amount of vasopressors, the blood pressure remains under 100 mm Hg systolic.

The learner then consults cardiology or a cardiothoracic surgeon for circulatory support options such as extracorporeal membrane oxygenation (ECMO) or an intra-aortic balloon pump. The patient is admitted to the ICU. The patient is placed on psychiatric hold and psychiatry is consulted to see the patient once awake. The learner explains everything to the mother and grandmother that arrived later, and they are sent to the ICU waiting room.

**Disposition:** Admission to Intensive Care Unit (ICU)

**Critical Actions:**

1. Orders chest compressions and atropine (patient care, medical knowledge)
2. Intubates patient (patient care)
3. Consults Poison Control and treats overdose – calcium, high-dose insulin, norepinephrine, lipid emulsion, methylene blue. (IP, medical knowledge, patient care, system-based practice)
4. Consults cardiology or cardiothoracic surgery for circulatory support options (patient care)
5. Consults intensivist and admits to ICU (patient care, IP)

### Historical Information

**Chief Complaint:** Altered mental status

**History of Present Illness:** The patient is a 29-year-old male, Mr. C, who is brought from home by the paramedics. He was found by his mother with pill bottles next to him. He was last seen well at 9 PM the prior evening. His mother went to check on him in the morning when he did not get up for work at 7 AM, and she was unable to wake him. The paramedics bring the pill bottles to the emergency department and the medication is amlodipine. The bottles were just filled three days ago and the prescription was for 90 days. The bottles are empty. Mother denies that the patient had had any recent illnesses. No further history is obtainable from the patient.

**Past Medical History:** Depression

**Past Surgical History:** Appendectomy

**Patient’s Medications:** None

**Allergies:** None

**Social history:**

- Smoking: Occasional
- Tobacco: None
- Drug use: None

**Family history:** Hypertension

### Physical Exam Information

[Table t2-jetem-9-1-o1]

**Weight:** 80.2 kg

**Primary survey:**

- **Airway:** Patent, no gag
- **Breathing:** Breath sounds present bilaterally but shallow
- **Circulation:** No palpable pulses, cool skin

**Physical examination:**

- **General appearance:** Unresponsive to all stimuli. Well-developed and well-nourished.
- **Head, eyes, ears, nose and throat (HEENT):**
  ○ **Head:** atraumatic, normocephalic
  ○ **Eyes:** Pupils equal and reactive
  ○ **Ears:** TMs are clear
  ○ **Nose:** Within normal limits
  ○ **Oropharynx/Throat:** Moist mucus membranes, pharynx clear, no cracked lips
- **Neck:** No lymphadenopathy, no thyromegaly, jugular vein distention (JVD) present
- **Chest:** Breath sounds present bilaterally, no wheezing, shallow
- **Cardiovascular:** Bradycardia, no murmurs
- **Abdominal/GI:** Decreased bowel sounds, soft, no hepatomegaly, no masses (cannot check tenderness)
- **Genitourinary:** Within normal limits
- **Rectal:** Within normal limits
- **Extremities:** No edema
- **Back:** Within normal limits
- **Neuro:** Unresponsive to all stimuli
- **Skin:** Cool and clammy, pale, no rashes
- **Lymph:** Within normal limits
- ● **Psych:** Unable to assess

### Critical Actions and Cueing Guidelines

1. **Critical Action 1:** *Orders chest compressions and atropine*
  (patient care, medical knowledge)
  This critical action is met by the learner telling the team to start chest compressions and ordering a milligram of atropine. The learner may repeat the atropine but there is no increase in the heart rate.
  Cueing guideline: If the learner does not order CPR or atropine, the nurse should say, “We are unable to measure a blood pressure, should we do something? I can’t imagine that he is perfusing at this rate and no blood pressure.”
2. **Critical Action 2:** *Intubates patient*
  (patient care)
  This critical action is met by the learner intubating the patient, checking for color change on the capnometer, listening to breath sounds bilaterally, and listening over the epigastrium. The learner may check the pulse oximeter but it remains low when compression are not being performed.
  Cueing guideline: If the learner does not intubate the patient, the oxygen saturation should continue to decrease despite 100% oxygen from 15L non rebreather.
3. **Critical Action 3:** *Consults Poison Control & treats overdose – calcium, high-dose insulin, norepinephrine, lipid emulsion, methylene blue.*
   (IP, medical knowledge, patient care)
  This critical action is met by the learner ordering calcium gluconate and epinephrine and then recognizing that the response only lasts for a few minutes. The learner then calls Poison Control for advice about treatment since calcium and epinephrine do not maintain the heart rate and the patient remains hypotensive. The learner then orders the high-dose insulin, norepinephrine, lipid emulsion, and methylene blue as recommended by Poison Control.
  Cueing guideline: If the learner does not treat the overdose, the nurse should say, “His blood pressure is still very low, is there anything else we can do?” The nurse may add, “The epi and the calcium seem to only last a few minutes. Are there other antidotes or treatment that might last longer?”
4. **Critical Action 4:** *Consults cardiology or cardiothoracic surgery for circulatory support options*
  (patient care)
  This critical action is met by the learner consulting either cardiology or cardiothoracic surgery for extracorporeal circulatory support options such as ECMO or intra-aortic balloon pump.
  Cueing guideline: If the learner does not initiate this, the nurse should prompt, “His blood pressure remains quite low; it does not seem that the medications are helping; is there anything else that can be done”

## ORAL BOARDS ASSESSMENT

*Calcium Channel Blocker Overdose*

Learner: _________________________________________

**Critical Actions:**

□ Orders chest compressions and atropine (patient care, medical knowledge)
□ Intubates patient (patient care)
□ Consults Poison Control & treats overdose – calcium, high-dose insulin, norepinephrine, lipid emulsion, methylene blue. (IP, medical knowledge, patient care, system-based practice)
□ Consults cardiology or cardiothoracic surgery for circulatory support options (patient care)
□ Consults intensivist and admits to ICU (patient care, IP)

**Summative and formative comments:**

**Milestone assessment:** [Table t3-jetem-9-1-o1]

### Stimulus Inventory

[Table t4-jetem-9-1-o1]

#### Stimulus #1

##### Patient Information

[Table t5-jetem-9-1-o1]

#### Stimulus #2

##### Rhythm Strip

[Fig f1-jetem-9-1-o1]

Image Source: Author’s own image.

#### Stimulus #3

##### 12 Lead EKG

[Fig f2-jetem-9-1-o1]

Image Source: Author’s own image.

#### Stimulus #4

##### Portable Chest X-ray

[Fig f3-jetem-9-1-o1]

Image Source: Gaillard F. Normal chest x-ray. Radiopaedia.org (Accessed on 29 Oct 2023) https://doi.org/10.53347/rID-8304

#### Stimulus #5

##### Portable Chest X-ray after Intubation

[Fig f4-jetem-9-1-o1]

Image Source: Normal CXR and Post-Intubation CXR. JETem (Accessed on 29 Oct 2023). https://jetem.org/ettcxr/

#### Stimulus #6

##### Complete Blood Count (CBC)

[Table t6-jetem-9-1-o1]

#### Stimulus #7

##### Chem 7 Basic Metabolic Panel (BMP)

[Table t7-jetem-9-1-o1]

#### Stimulus #8

##### Cardiac Enzymes

[Table t8-jetem-9-1-o1]

#### Stimulus #9

##### Venous Blood Gas and Lactic Acid

[Table t9-jetem-9-1-o1]

#### Stimulus #10

##### Alcohol, Salicylate, and Acetaminophen Levels

[Table t10-jetem-9-1-o1]

#### Stimulus #11

##### Urine Drug Screen

[Table t11-jetem-9-1-o1]

## DEBRIEFING AND EVALUATION PEARLS

### Calcium Channel Blocker Overdose

- CCB overdoses are life-threatening via their effect on the cardiovascular system, mainly myocardial depression and peripheral vasodilation which results in hypotension and bradycardia. Patients can develop both vasoplegic and cardiogenic shock.
- It is important to know if the patient took an extended release formulation of the drug because the manifestations can be delayed and prolonged. Pediatric overdoses are especially concerning. A single pill of nifedipine or verapamil could kill a child.
- Workup may reveal: hypokalemia, hyperglycemia, and evidence of poor perfusion such as elevated lactic acid.^1^
- There are many proposed treatments for CCB overdose with variable evidence to support them. If the patient is critically ill, it is advised to try a multiple of therapies at the same time.
- CCB should be treated with IV fluid, calcium supplementation, high dose insulin, and vasopressors. Pacing, lipid emulsion therapy, and circulatory support options can be considered for refractory cases.^3^
